# Dietary supplementation with *N*-acetyl-L-cysteine ameliorates hyperactivated ERK signaling in the endometrium that is linked to poor pregnancy outcomes following ovarian stimulation in pigs

**DOI:** 10.1186/s40104-024-01109-1

**Published:** 2024-11-06

**Authors:** Linghua Cheng, Zhicheng Shi, Yuan Yue, Yue Wang, Yusheng Qin, Wei Zhao, Yupei Hu, Qin Li, Min Guo, Lei An, Shumin Wang, Jianhui Tian

**Affiliations:** Frontiers Science Center for Molecular Design Breeding (MOE), State Key Laboratory of Animal Biotech Breeding, Key Laboratory of Animal Genetics, Breeding and Reproduction of the Ministry of Agriculture and Rural Affairs, National Engineering Laboratory for Animal Breeding, College of Animal Science and Technology, China Agricultural University, Beijing, 100193 People’s Republic of China

**Keywords:** Estradiol, Gonadotropin, Implantation, *N*-acetyl-L-cysteine, Ovarian stimulation, Uterine receptivity

## Abstract

**Background:**

Exogenous gonadotropin-controlled ovarian stimulation is the critical step in animal reproductive management, such as pig, sheep, bovine and other species. It helps synchronize ovulation or stimulate multiple ovulations. However, a number of evidence indicated an unexpected decrease in pregnancy outcomes following ovarian stimulation. This study aimed to explore the underlying mechanism of the pregnancy defect and develop a practical rescue strategy.

**Results:**

Compared with those in the control group, gilts that underwent ovarian stimulation showed a decrease in pregnancy rate, farrowing rate, and total number of piglets born. Stimulated gilts also showed an increase in estradiol (E_2_) levels. The supraphysiological E_2_ level was correlated with the decrease in the number of piglets born. Furthermore, we found that high levels of E_2_ impair uterine receptivity, as shown by the overproliferation of endometrial epithelial cells. In vitro mechanistic studies demonstrated that high levels of E_2_ hyperactivate FGF-FGFR-ERK signaling cascade in the uterine endometrium, and in turn induces overproliferation of endometrial epithelial cells. Of note, *N*-acetyl-L-cysteine (NAC) supplementation effectively inhibits ERK hyperphosphorylation and ameliorates endometrial epithelial overproliferation. Importantly, in vivo experiments indicated that dietary NAC supplementation, compared with ovarian stimulation group, improves the uterine receptivity in gilts, and significantly increases the pregnancy rate and total number of piglets born.

**Conclusions:**

Ovarian stimulation-induced supraphysiological levels of E_2_ impairs uterine receptivity by hyperactivating FGF-FGFR-ERK signaling cascade, thereby reducing pregnancy rate and litter size. Supplementing NAC to a conventional diet for gilts ameliorates hyperactivated ERK signaling and improves uterine receptivity, thus rescuing adverse pregnancy outcomes following ovarian stimulation.

**Graphical Abstract:**

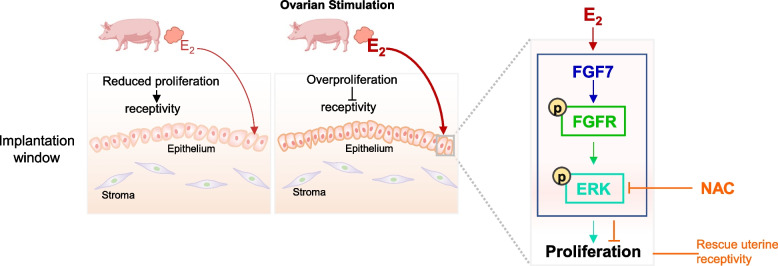

**Supplementary Information:**

The online version contains supplementary material available at 10.1186/s40104-024-01109-1.

## Introduction

Exogenous gonadotropin-controlled ovarian stimulation is the critical step and has been extensively used not only in animal reproductive management, but also in human assisted reproduction. In animal reproductive management, exogenous gonadotropin not only induces multiple ovulations, but also makes synchronization of ovulation and fixed-time artificial insemination (FTAI) feasible. In particular, this is of great beneficial for intensive pig farming, because it allows the practice of “all-in-all-out” and the production of large groups of pigs in the same reproductive state with the same health and immunization status, which is also referred to as batch management [1]. In standard in vitro fertilization (IVF) and embryo transfer programs, exogenous gonadotropins are used alone or in combination to stimulate the growth and maturation of multiple follicles, which provides the opportunity for retrieving a greater number of oocytes, and in turn allows more embryos to be transferred [2].

Despite the importance and benefit of exogenous gonadotropin-controlled ovarian stimulation, many animal and human studies have shown unfavorable outcomes, including impaired implantation and post-implantation embryonic loss in mice [2, 3] and rats [4], as well as increased implantation failure and lower birth weight in humans [5, 6]. In stimulated gilts or sows, although the ovulation rate is increased by treatment of pregnant mare serum gonadotrophin (PMSG, also called equine chorionic gonadotropin), a decreased embryonic survival rate and lower pregnancy rate [7–9] have been reported as early as the 1960s. The high rate of implantation failure and embryonic loss was considered the main reason for the major cause of reduced litter size [8–11], a significant obstacle that hinders commercial benefits of FTAI in the past several decades.

So far, however, the underlying mechanisms responsible for reduced fertility and adverse pregnancy outcomes following ovarian stimulation, have not been fully understood in either humans or animals. One of the most convincing and direct reasons is disrupted ovarian steroidogenesis [3, 7, 12], but the downstream signaling mechanism has never been determined. Moreover, although several deleterious defects, such as poor oocyte quality and a compromised embryonic developmental potential, have been linked to the unsatisfactory outcomes [13, 14], the critical contributing factor remains controversial and functional evidence is lacking. Even though, among the potential factors, increasing evidence from mouse models [15, 16], human clinical practice [6], and stimulated sows [17], indicates that reduced implantation rates associated with ovarian stimulation may be due to an impairment of uterine receptivity.

Here, we hypothesized that exogenous gonadotropin-controlled ovarian stimulation disrupts ovarian steroidogenesis, thus impairing uterine receptivity, which in turn results in the pregnancy defects. In the present study, we aimed to investigate the mechanism responsible for the unsatisfactory pregnancy outcomes after ovarian stimulation in pigs, and develop the promising rescue strategy based on our mechanistic exploration.

## Materials and methods

All animal studies were conducted in accordance with the China Agricultural University Institutional Animal Care and Use Committee.

### Exp. 1: The effect of ovarian stimulation on steroidogenesis and pregnancy outcomes in gilts

#### Animals and experimental design

**Fig. 1:**
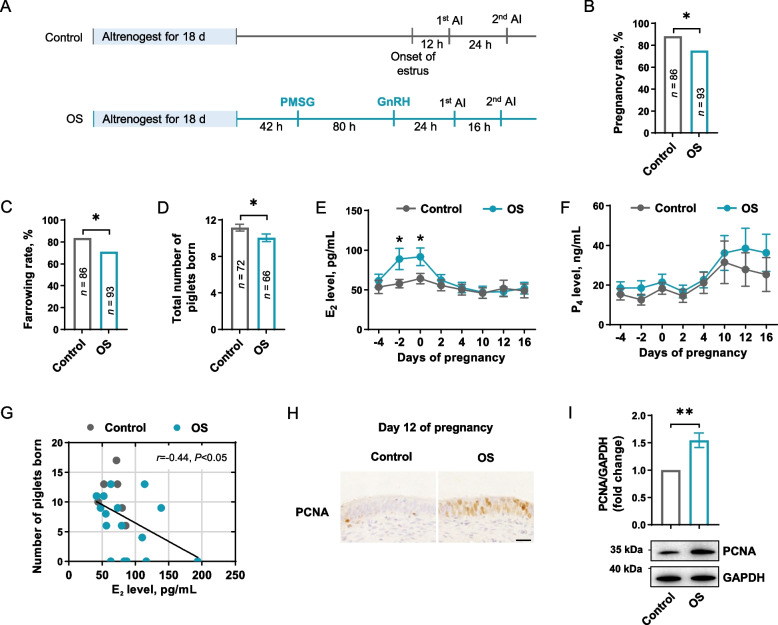
Ovarian stimulation triggers supraphysiological E_2_ levels and results in a decreased in pregnancy rate and litter size. **A** Schematic illustration of experimental design where gilts were treated with or without ovarian stimulation before FTAI. **B–D** The pregnancy rate (**B**), farrowing rate (**C**) and litter size (**D**) in gilts treated with (OS) or without (Control) ovarian stimulation. **E** and **F** The serum levels of E_2_ (**E**) and P_4_ (**F**) in control and OS groups. **G** Correlation between serum E_2_ levels and the number of piglets born, each dot represents a gilt. **H** Immunohistochemical analysis of PCNA in endometrium from gilts on d 12 of pregnancy in control and OS groups. Scale bar: 20 μm. **I** Western blotting analysis of PCNA in endometrium from gilts on d 12 of pregnancy in control and OS groups (*n* = 3). The upper panel shows the quantification of PCNA level. Data are presented as the mean ± SEM. *n* value represents the number of gilts. ^*^P < 0.05, ^**^P < 0.01. OS, ovarian stimulation; PMSG, pregnant mare serum gonadotrophin; GnRH, gonadotropin-releasing hormone; AI, artificial insemination

This experiment was conducted with a total of 188 gilts (Landrace × Yorkshire, initial body weight: 120–140 kg) at 8 months of age. Gilts were housed in pens in an environmentally controlled room with the temperature maintained at 23 ± 2 °C, and the humidity at 60%–70%. Water and feed were available to the gilt ad libitum. To test the effect of ovarian stimulation on steroidogenesis and pregnancy outcomes, gilts were randomly divided into two groups (Fig. 1A): control (*n* = 95) and ovarian stimulation (OS, *n* = 93). In the control group, gilts were synchronized for estrus by daily oral administration of 20 mg/gilts of altrenogest (Ningbo Sansheng, Zhejiang, China) for 18 d. Then, the estrus of gilts was detected in the presence of the boar by observing the standing reflex of gilts in response to back pressure twice daily from d 3 to 10 after altrenogest was withdrawn. Artificial insemination was performed twice at 12 h and 24 h after the onset of estrus in 86 gilts exhibiting standing estrus. In the OS group, gilts were synchronized for estrus with altrenogest, similar to the gilts in the control group. And then, gilts received an intramuscular (IM) injection of 1,000 IU PMSG (Ningbo Sansheng) at 42 h after the last administration of altrenogest, followed by an IM injection of 100 µg gonadotropin-releasing hormone (GnRH) analogues (Ningbo Sansheng) at 80 h after PMSG administration. Thereafter, all gilts were inseminated twice at 24 h after the GnRH administration and 16 h after the first FTAI, regardless of estrus expression. On d 12 of pregnancy, gilts from control and OS group were slaughtered via exsanguinations with anesthesia, the conceptuses and endometrial tissue were collected. The pregnancy rate, farrowing rate and total number of piglets born per litter were evaluated.

#### Blood collection and hormone analysis

The blood samples were collected from each gilt’s precaval vein in vacutainer tubes on the day of injection of PMSG, then every 2 d including the first day of estrus and subsequently on d 2, 4, 10, 12 and 16 after FTAI. Then, the blood samples were centrifuged at 3,000 × *g* for 30 min at 4 °C, and serum samples were isolated by carefully collecting the supernatant. The serum samples were then stored at −20 °C until hormone measurement. The serum E_2_ levels were measured by radioimmunoassay as described previously [18].

### Exp. 2: The mechanism responsible for the poor pregnancy outcomes following ovarian stimulation

To understand how ovarian stimulation induces poor pregnancy outcomes, we used an in vivo pubertal mouse model mimicking ovarian stimulation and an in vitro cultured porcine endometrial explant model, to investigate the mechanism responsible for the poor pregnancy outcomes.

#### Experimental design of in vivo mouse model

A total of 85 2-month-old ICR female mice (initial body weight: 25–35 g) was randomly divided into two groups: control (*n* = 35) and OS (*n* = 50). In the control group, female mice exhibiting estrus were co-caged individually with fertile males. In the OS group, mice were treated with intraperitoneal (IP) injection of 5 IU PMSG (Ningbo Sansheng) and a further 5 IU human chorionic gonadotropin (hCG; Ningbo Sansheng) 46–48 h later. Then, female mice were co-caged individually with fertile ICR males. In both the control group and the OS group, the vaginal plug of female mice was examined on the second day after mating. The presence of vaginal plugs was considered as an indication of successful mating, and this day was designated d 1 of pregnancy.

For the embryo donation mouse model, 8 female mice were used as stimulated donors, and 9 2-month-old ICR female mice (initial body weight: 25–35 g) were randomly divided into two recipient mice groups (Fig. S3D): unstimulated recipients (*n* = 4) and stimulated recipients (*n* = 5). The treatment of stimulated donors is as described for the OS group above. The stimulated recipients also received PMSG and hCG injections, while the unstimulated recipients were injected with a saline solution. Then, pseudopregnant recipients were prepared by mating with vasectomized male mice. On d 4 of pregnancy, the donor mice were sacrificed via cervical dislocation, and embryos were retrieved from the uteri of donor mice. Then 6 embryos were transferred to each uterine horn of recipient mice in different groups, respectively. The number of corpora lutea (CL) was counted to quantify the number of ovulated oocytes, and embryo implantation was detected on d 5 by intravenous injection of 0.1 mL of 0.1% Chicago blue dye (Sigma, Burlington, MA, USA) before sacrificing [19].

#### Experimental design of in vitro cultured porcine endometrial explants model

Porcine endometrial explants were used as the in vitro model to test the impact of high levels of E_2_ on uterine receptivity, as well as implicated pathways. The endometrial explants were cultured as described previously [20]. Briefly, uteri were obtained from gilts on d 5–8 of the estrous cycles at an abattoir and then transported to the laboratory on ice. After washing with sterile saline solution containing penicillin–streptomycin, the endometrium was dissected from the myometrium and cut into small pieces (2–3 mm^3^). Endometrial explants were cultured in DMEM/F-12 medium (Invitrogen) with 10 μg/mL insulin (Sigma), 10 ng/mL transferrin (Sigma), 10 ng/mL hydrocortisone (Abcam, Cambridge, CB2 0AX, UK), 100 units/mL penicillin, 100 µg/mL streptomycin and 0.25 µg/mL Amphotericin B (both from Solarbio, Beijing, China) at 37 °C with 5% CO_2_.

**Fig. 2:**
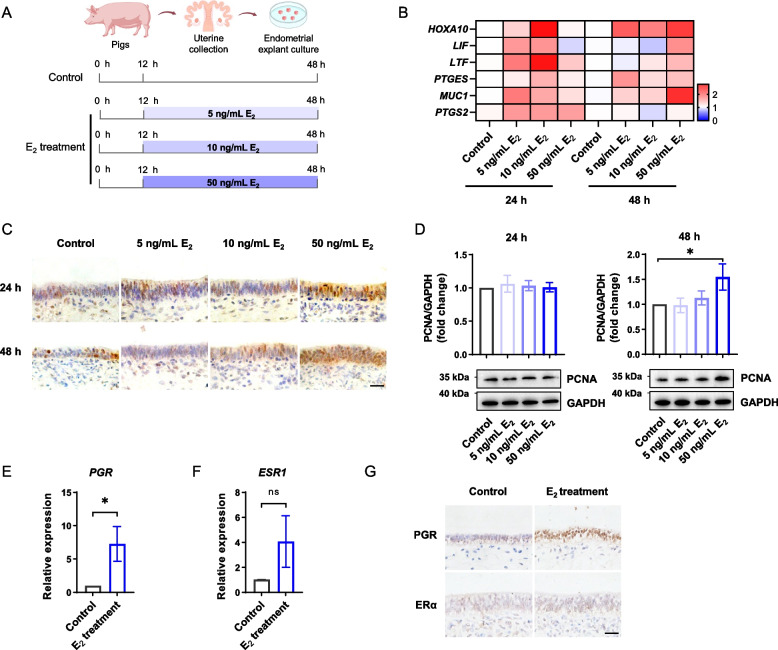
Supraphysiological E_2_ impairs endometrial receptivity. **A** Schematic illustration of experimental design where endometrial explants were treated with different concentrations of E_2_. **B** Heatmap showing the relative mRNA expression levels of genes associated with endometrial receptivity in endometrial explants treated with different concentrations of E_2_ or 24 h (*n* = 6) and 48 h (*n* = 5). Colored bars indicate relative mRNA expression levels detected by qPCR. **C** Immunohistochemical analysis of PCNA in endometrial explants treated with different concentrations of E_2_ for 24 h and 48 h. Scale bar: 20 μm. **D** Western blotting analysis of PCNA in endometrial explants treated with different concentrations of E_2_ for 24 h (*n* = 6) and 48 h (*n* = 5). The upper panel shows the quantification of PCNA level. **E** and **F** Relative mRNA levels of *PGR* (**E**) and *ESR1* (**F**) in endometrial explants treated with or without the high level of E_2_ (*n* = 5). **G** Immunohistochemical analysis of PGR and ER in endometrial explants treated with or without E_2_. Scale bar: 20 μm. Data are presented as the mean ± SEM. ^*^P < 0.05, ^**^P < 0.01. ns, not significant. E_2_, estradiol; PCNA, proliferating cell nuclear antigen; *PGR*, progesterone receptor; *ESR1*, estrogen receptor 1

Endometrial explants were cultured in medium supplemented with E_2_ (Sigma) at different concentrations (5, 10, 50 ng/mL) or without E_2_ (control group) for 24 or 48 h (Fig. 2A). To further investigate the downstream pathways, explants were cultured in the presence of 200 ng/mL of recombinant fibroblast growth factor 7 (FGF7; Invitrogen), 10 μmol/L FGFR inhibitor BGJ398 (Selleck, Houston, USA), or 100 nmol/L ERK inhibitor PD0325901 (Selleck). The endometrium explants were sampled from at least three individual gilts, and treatments were performed in duplicate using endometrial explants obtained from each gilt. After treatment, endometrial explants were collected for histological and molecular analyses.

#### RNA extraction and quantitative real-time PCR analysis

Total RNA was extracted from endometrial explants using TRIzol™ (Invitrogen, Carlsbad, CA, USA) following the manufacturer’s instruction. RNA quality and quantity were measured with DeNovix DS-11 fluorometer (DeNovix Inc., Delaware, USA). The integrity of RNA was assessed by the ratio of 28S and 18S ribosomal RNA band following 1% agarose gel electrophoresis. Then, 1 μg total RNA was treated with DNase I to remove genomic DNA, and reverse-transcribed into cDNA using the HiScript II Q Select RT Kit (Vazyme, Nanjing, China) according to the manufacturer’s instruction. Primers used in this experiment were designed with Oligo 7 (Molecular Biology Insights, Inc., Colorado Springs, CO, USA) and are listed in Table S1. The quantitative real-time PCR analysis (qPCR) was performed using SsoFast EvaGreen Supermix (Bio-Rad, Hercules, CA, USA) on the CFX96 Real-Time PCR system (Bio-Rad). The qPCR reaction contained 5 μL of 2 × SsoFast™ EvaGreen^®^ Supermix, 3 μL of RNAase-free water, 1 μL of cDNA sample and 1 μL of primers. The qPCR program was performed as follows: denaturation at 95 °C for 30 s, 40 cycles of 95 °C for 5 s, and 60 °C for 5 s. Efficiency of the qPCR for both target and reference genes were between 90% and 110%, corresponding to a slope from −3.0 to −3.4 and *R*^2^ of calibration curve were above 0.95. In addition to this assessment, PCR was performed using a no reverse transcriptase control template, which was treated with DNase I but without the reverse transcription step, and primers overlap exon-exon borderlines was used to eliminate the absence of any genomic DNA contamination. To choose the most stable reference gene, 8 candidate reference genes were tested in samples under different treatments, and the stability of these genes was confirmed using NormFinder software as described previously. The relative messenger RNA (mRNA) expression level were calculated using the 2^−△△Cq^ method. The relative mRNA expression level was normalized to the reference gene Glyceraldehyde-3-phosphate dehydrogenase (*GAPDH*), and expressed as fold change relative to the control. To quickly and easily visualize patterns of gene expression that are associated with the concentration and duration of E_2_ exposure, the data were converted into a heatmap using GraphPad Prism version 9.0.0. Each row represents a gene and each column represents a treatment condition. Red represents upregulation, and blue represents downregulation, color intensity represents the relative mRNA expression level.

#### Immunohistochemical analysis

Endometrial explants were fixed with 4% paraformaldehyde at 4 °C for 48 h. Fixed explants were sectioned at 5 µm using vibratome (VT1000S; Leica, Wetzlar, Germany), and then deparaffinized and rehydrated. The slides were boiled in citrate unmasking solution and cooled to room temperature (RT), washed with phosphate buffered saline (PBS), and incubated with 3% hydrogen peroxide for 10 min. After blocking with PBS containing 0.5% bovine serum albumin for 45 min at RT, the slides were incubated with the primary mouse anti-proliferating cell nuclear antigen (PCNA) antibody (1:200; Abcam), rabbit anti-estrogen receptor alpha (ERα) antibody (1:400; Abcam), rabbit anti-progesterone receptor (PGR) antibody (1:400; Proteintech, Rosemont, IL, USA), rabbit anti-FGF7 antibody (1:200; Abcam), or rabbit anti-phosphorylated ERK (pERK) antibody (1:100; Cell Signaling Technology) overnight at 4 °C. As a negative control, species matched IgG was used to prevent non-specific binding (Fig. S1). Slides were then incubated with horseradish peroxidase (HRP) conjugated anti-rabbit or anti-mouse IgG (1:200; Zhongshan Jinqiao, Beijing, China) for 1 h at RT, and then washed again. Detection was performed using the 3,3′-diaminobenzidine reaction (Zhongshan Jinqiao). Images were captured under an upright microscope (BX51; Olympus) using an attached digital microscope camera (DP72; Olympus), and at least three images containing regions of endometrial epithelium were randomly captured from each section. The antibodies used were shown in Table S2.

#### Protein extraction and Western blotting

Whole protein extracts were prepared by incubating endometrial explants with lysis buffer for 30 min on ice. The protein concentration was measured by Bicinchoninic Acid assay (Beyotime Biotechnology, Shanghai, China). Then the protein was separated on 12% acrylamide gels containing 0.1% sodium dodecyl sulfate and transferred onto hydrophobic polyvinylidene fluoride membranes (Millipore, Billerica, MA, USA). The membranes were blocked with 5% non-fat dried milk in Tris buffered saline (TBS) containing 0.1% Tween-20 for 1 h at RT and then incubated overnight at 4 °C with diluted antibodies: mouse anti-PCNA antibody (1:1,000; Abcam), rabbit anti-FGF7 antibody (1:1,000; Abcam), rabbit anti-phosphorylated FGFR (pFGFR) antibody (1:1,000; Cell Signaling Technology, Danvers, MA, USA), rabbit anti-pERK antibody (1:1,000; Cell Signaling Technology), rabbit anti-ERK antibody (1:1,000; Cell Signaling Technology) and mouse anti-GAPDH antibody (1:1,000; Abcam), followed by three washes with TBS containing 0.1% Tween-20 for 45 min. As a negative control, species matched IgG was used as the primary antibody to prevent non-specific binding (Fig. S2). HRP conjugated anti-rabbit or anti-mouse IgG (1:5,000; Zhongshan Jinqiao, Beijing, China) were incubated for 1.5 h, and protein bands were detected using Tanon 5200 detection systems (Tanon, Shanghai, China). Details of the antibodies used were shown in Table S2.

### Exp. 3: The effect of dietary NAC supplementation on embryo development and pregnancy outcomes following ovarian stimulation

#### Animals and experimental design

This experiment was conducted with a total of 689 gilts (Landrace × Yorkshire, initial body weight: 120–140 kg) at 8 months of age. Gilts were housed in pens in an environmentally controlled room. Water and feed were available to the gilt ad libitum. The stock solution (100 g/L) of NAC (Yuancheng Gongchuang Technology Co. Ltd., Wuhan, China) was prepared in water, then the NAC solution or an equal volume of water (control diet) was added to the basic diet. Gilts that underwent standard ovarian stimulation were randomly divided into four groups. In each group, stimulated gilts were fed a control diet (OS, *n* = 175) or a diet supplemented with different concentrations of NAC (OS + 1 g/kg NAC, *n* = 159; OS + 3 g/kg NAC, *n* = 206; OS + 9 g/kg NAC, *n* = 149) from altrenogest withdrawal to the post-implantation stage (d 28 of pregnancy). The pregnancy rate, farrowing rate, and total number of piglets born per litter, were evaluated at indicated time points (Fig. 6A). On d 12 of pregnancy, 7 gilts from OS group and 6 gilts from OS + 3 g/kg NAC group were slaughtered respectively via exsanguinations with anesthesia. Then, 12 gilts (OS, *n* = 6; OS + 3 g/kg NAC, *n* = 6) were slaughtered on d 17 of pregnancy. The number of ovulated oocytes was determined by counting the number of CL from both ovaries of each gilt, referring to the previously published studies [21, 22], and conceptuses were collected by flushing the uterine horn. Conceptuses showing tubular and filamentous forms on d 12 of pregnancy, as well as those elongated viable conceptuses on d 17 of pregnancy, are considered as morphologically normal conceptuses, as previously described [23, 24]. In contrast, other forms are defined as morphologically abnormal conceptus.

For embryo donation mouse model, a total of 93 2-month-old ICR female mice (initial body weight: 25–35 g) were used as recipients and randomly divided into three groups: untreated recipients (*n* = 61), recipients that underwent standard ovarian stimulation (*n* = 16), and stimulated recipients that received NAC treatment (*n* = 16). Furthermore, 80 2-month-old ICR female mice (initial body weight: 25–35 g) were used as donors and randomly divided into two groups: stimulated donors (*n* = 50) and stimulated donors that received NAC treatment (*n* = 30). The donors and recipients from same experiment were mated at the same time. For stimulated donors or recipients that received NAC treatment, mice were intraperitoneally injected with 0.1 mL of 45 mg/mL NAC on d 3 before PMSG administration, and once daily for 10 d. For stimulated control recipients or donors, mice were intraperitoneally injected with an equivalent volume of physiological saline. The stimulated mice received an IP injection of PMSG, followed by an IP injection of hCG and were mated with fertile or vasectomized male mice, respectively. The untreated recipients were mated with vasectomized males during estrus. The day of vaginal plugs appearance was designated d 1 of pregnancy. On d 4 of pregnancy, the donor mice were sacrificed via cervical dislocation, and embryos were recovered from the uteri of donor mice. Then embryos were transferred to the uterine horn of recipient mice in different group, respectively.

### Statistical analysis

Statistical analyses were performed using SPSS v.25.0 (IBM, Armonk, NY, USA). Before analysis, the normal distribution of data was validated using the Kolmogorov–Smirnov test. For the analysis of gene and protein expression levels, hormone levels, the total number of piglets born, and the number of ovulated oocytes or conceptuses, data were analyzed with the student’s *t*-test. For the analysis of the pregnancy rate and farrowing rate, the Chi-square test was used. The relationship of preovulatory E_2_ levels and number of piglets born were analyzed by Spearman correlation analysis. Data are presented as mean ± standard error of the mean (SEM). Values of *P* < 0.05 were considered statistically significant.

## Results

### Exp. 1

#### Ovarian stimulation triggers supraphysiological E_2_ levels that are associated with reduced pregnancy rate and lower litter size

To understand the mechanism underlying ovarian stimulation-associated unfavorable pregnancy outcomes in pigs, we first detected the correlation between steroidogenesis and pregnancy outcomes. By comparing standard and well-established FTAI procedures for gilts with or without gonadotropin treatment (Fig. 1A), we found that the ovarian stimulation regimen resulted in a decrease in both pregnancy rate and farrowing rate (*P* < 0.05, Fig. 1B and C), as well as a reduced litter size at farrowing (*P* < 0.05, Fig. 1D). Then, we performed a time-course detection of E_2_ and progesterone (P_4_) from the preovulatory to peri-implantation stage. The results indicated that ovarian stimulation led to supraphysiological levels of E_2_ during the preovulatory phase (*P* < 0.05, Fig. 1E), but did not affect progesterone levels (*P* > 0.05, Fig. 1F). More importantly, the hyperestrogenic state was tightly correlated with reduced litter size (*P* < 0.05, Fig. 1G). In addition, we detected the proliferation of endometrium on d 12 of pregnancy, at which restrictedly regulated proliferation is essential for initial adhesion and thus is essential for successful implantation. We found an endometrial overproliferation that is regionally restricted to epithelium (Fig. 1H). Western blotting analysis further confirmed the increased proliferation in OS group (*P* < 0.05, Fig. 1I).

### Exp. 2

#### Ovarian stimulation-induced supraphysiological E_2_ levels are linked to impaired uterine receptivity

We next attempted to understand how ovarian stimulation impaired fertility. To facilitate the detection of developmental defects that occur in stimulated gilts, we used the stimulated pubertal mouse model that recapitulates the increased ovulation rate and decreased pregnancy outcomes following ovarian stimulation (*P* < 0.01, Fig. S3A and B). Despite the increase in ovulated oocytes, ovarian stimulation led to a much lower chance of successful implantation suggesting that ovarian stimulation has a prolonged adverse effect on subsequent implantation (*P* < 0.01, Fig. S3C). To exclude the possible effect of the quality of preimplantation embryos on these results, we next used a well-established embryo donation model (Fig. S3D) [15]. Our results indicated that changes in the uterine environment, rather than embryo developmental potential per se, should be responsible for implantation failure associated with ovarian stimulation (*P* < 0.01, Fig. S3E).

To establish the direct functional link between high levels of ovarian E_2_ and impaired uterine environment, we used a well-established in vitro model of endometrial explants from mature gilts (Fig. 2A). After being exposed to various concentrations of E_2_ for 24 h or 48 h, endometrial explants showed notable changes in the mRNA levels of genes associated with uterine receptivity, albeit to varying degrees (Fig. 2B). Of note, products of genes that regulate endometrial proliferation during the establishment of endometrial receptivity, i.e., homeobox A10 (*HOXA10*) and mucin 1 (*MUC1*), were dysregulated in response to high levels of E_2_ (*P* < 0.05, Fig. 2B). Immunohistochemical analysis of PCNA showed that E_2_ primarily stimulated the proliferation of epithelium (Fig. 2C). Meanwhile, Western blotting analysis showed that E_2_ stimulated epithelium proliferation in a time- and dose-dependent manner. A relatively long exposure (48 h) to high concentrations of E_2_ (50 ng/mL) resulted in a significant increase in epithelium proliferation (*P* < 0.05, Fig. 2D). Thus, this concentration was used in the following experiments. Furthermore, we found that a high concentration of E_2_ (50 ng/mL) significantly upregulated the mRNA levels of *PGR* in endometrial explants (Fig. 2E and F). Immunohistochemical analysis revealed that ER and PGR were primarily localized in the epithelium (Fig. 2G).

#### The high level of E_2_ induces overproliferation of endometrial epithelial cells by hyperactivating FGF-FGFR-ERK signaling cascade

**Fig. 3:**
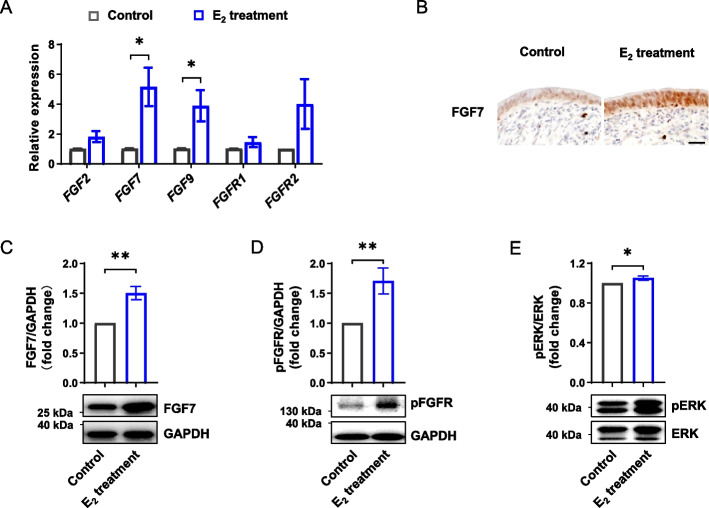
The high level of E_2_ hyperactivates FGF-FGFR-ERK signaling cascade. **A** Relative mRNA expression levels of *FGFs* and their receptors in endometrial explants treated with or without the high level of E_2_ (*n* = 5). **B** Immunohistochemical analysis of FGF7 in endometrial explants treated with or without the high level of E_2_. Scale bar: 20 μm. **C** Western blotting analysis of FGF7 level in endometrial explants treated with or without the high level of E_2_ (*n* = 7). The upper panel shows the quantification of FGF7 protein level. **D** and **E** Western blotting analysis of phosphorylation levels of FGFR (**D**) and ERK (**E**) in endometrial explants treated with or without the high level of E_2_ (*n* = 7). The upper panel shows the quantification of pFGFR and pERK levels. Data are presented as the mean ± SEM. ^*^P < 0.05, ^**^P < 0.01. E_2_, estradiol; *FGF*, fibroblast growth factor; FGF7, fibroblast growth factor 7; FGFR, fibroblast growth factor receptor; ERK, extracellular signal-regulated kinase; pFGFR, phosphorylated FGFR; pERK, phosphorylated ERK

We next attempted to understand the mechanism responsible for the endometrial overproliferation induced by supraphysiological E_2_. We found that mRNA levels of *FGF7* and *FGF9* were significantly increased in endometrial explants due to exposure to the high level of E_2_ (Fig. 3A). In particular, FGF7 showed a significant upregulation in mRNA levels (*P* < 0.05, Fig. 3A) and robust increase in the protein level in the epithelium (*P* < 0.01, Fig. 3B and C) due to exposure to the high level of E_2_. As expected, downstream FGFR-ERK signaling was, in turn, hyperactivated (*P* < 0.05, Fig. 3D and E).

**Fig. 4:**
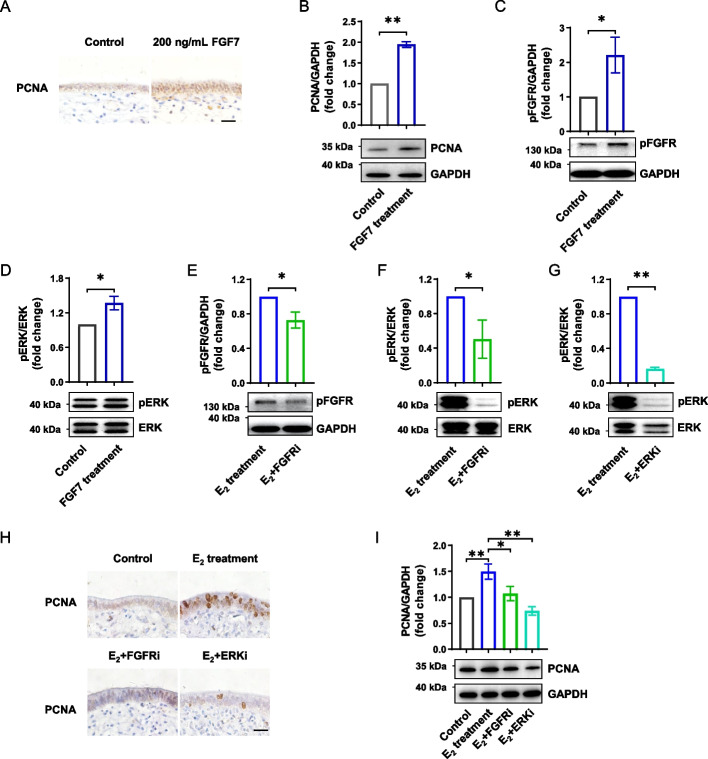
The high level of E_2_ induces overproliferation of endometrium via FGF-FGFR-ERK signaling cascade. **A** Immunohistochemical analysis of PCNA in endometrial explants treated with or without recombinant FGF7. Scale bar: 20 μm. **B** Western blotting analysis of PCNA in endometrial explants treated with or without recombinant FGF7 (*n* = 7). The upper panel shows the quantification of PCNA level. **C** and **D** Western blotting analysis of phosphorylation levels of FGFR (**C**) and ERK (**D**) in endometrial explants treated with or without recombinant FGF7 (*n* = 7). The upper panels show the quantification of pFGFR and pERK levels. **E** and **F** Western blotting analysis of phosphorylation levels of FGFR (**E**) and ERK (**F**) in endometrial explants treated with the high level of E_2_ alone or in combination with FGFR inhibitor (*n* = 5). The upper panels show the quantification of pFGFR and pERK levels. **G** Western blotting analysis of phosphorylation levels of ERK in endometrial explants treated with the high level of E_2_ alone or in combination with ERK inhibitor. The upper panel shows the quantification of the pERK level (*n* = 5). **H** Immunohistochemical analysis of PCNA in endometrial explants treated with or without FGFR inhibitor or ERK inhibitor. Scale bar: 20 μm. **I** Western blotting analysis of PCNA in endometrial explants treated with or without FGFR inhibitor or ERK inhibitor (*n* = 5). The upper panel shows the quantification of PCNA level. Data are presented as the mean ± SEM. ^*^P < 0.05, ^**^P < 0.01. PCNA, proliferating cell nuclear antigen; FGF7, fibroblast growth factor 7; FGFR, fibroblast growth factor receptor; ERK, extracellular signal-regulated kinase; pFGFR, phosphorylated FGFR; pERK, phosphorylated ERK; E_2_, estradiol

To further confirm the role of the FGF-FGFR-ERK signaling cascade in endometrial overproliferation induced by high levels of E_2_, we directly treated the endometrial explants with various concentrations of exogenous FGF7. We found 200 ng/mL FGF7 supplementation recapitulated endometrial epithelium overproliferation (*P* < 0.01, Fig. 4A and B) and hyperactivated FGFR-ERK signaling (*P* < 0.05, Fig. 4C and D), similar to those induced by high level of E_2_. To functionally determine whether endometrial overproliferation depends on FGFR-ERK signaling, we next blocked FGFR and ERK using BGJ398 and PD0325901, respectively. When phosphorylated activation of either FGFR or ERK was inhibited (Fig. 4E–G), endometrial overproliferation induced by high level of E_2_, was reduced to a level comparable to that in the control group (*P* < 0.01, Fig. 4H and I).

#### NAC inhibits hyperactivation of ERK signaling and alleviates endometrial overproliferation

**Fig. 5:**
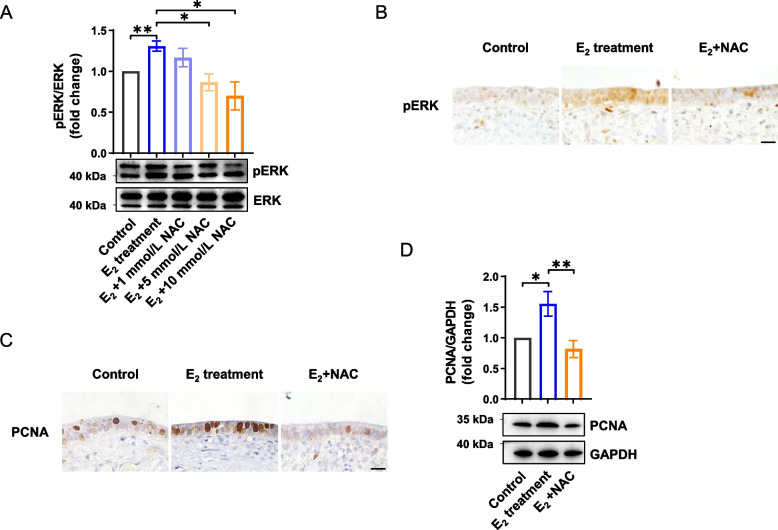
NAC alleviates endometrial overproliferation by inhibiting hyperactivation of FGF-FGFR-ERK signaling cascade. **A** Western blotting analysis of phosphorylation levels of ERK in endometrial explants treated with different concentrations of NAC (*n* = 4). The upper panel shows the quantification of the pERK level. **B** Immunohistochemical analysis of phosphorylation levels of ERK in endometrial explants treated with or without NAC. Scale bar: 20 μm. **C** Immunohistochemical analysis of PCNA in endometrial explants treated with or without NAC. Scale bar: 20 μm. **D** Western blotting analysis of PCNA in endometrial explants treated with or without NAC (*n* = 5). The upper panel shows the quantification of PCNA level. Data are presented as the mean ± SEM. ^*^P < 0.05, ^**^P < 0.01. ERK, extracellular signal-regulated kinase; pERK, phosphorylated ERK; NAC, *N*-acetyl-L-cysteine; PCNA, proliferating cell nuclear antigen

We next attempted to screen modulators that could ameliorate FGFR-ERK hyperphosphorylation. Among selected candidates, we noticed NAC, the acetylated variant of the amino acid L-cysteine, is a safe and well-tolerated medication that has been widely used worldwide in various pharmacological applications [25]. Next, we used the endometrial explant model, to determine if NAC could alleviate endometrial overproliferation when exposed to high levels of E_2_, we found that NAC supplementation inhibited ERK hyperactivation induced by the high level of E_2_, in a dose dependent manner (*P* < 0.05, Fig. 5A and B). In line with this, 10 mmol/L NAC supplementation significantly inhibited endometrial epithelial overproliferation to a level comparable to that in the control group (*P* < 0.01, Fig. 5C and D).

### Exp. 3

#### Dietary NAC supplementation improves pregnancy outcomes following ovarian stimulation

**Fig. 6:**
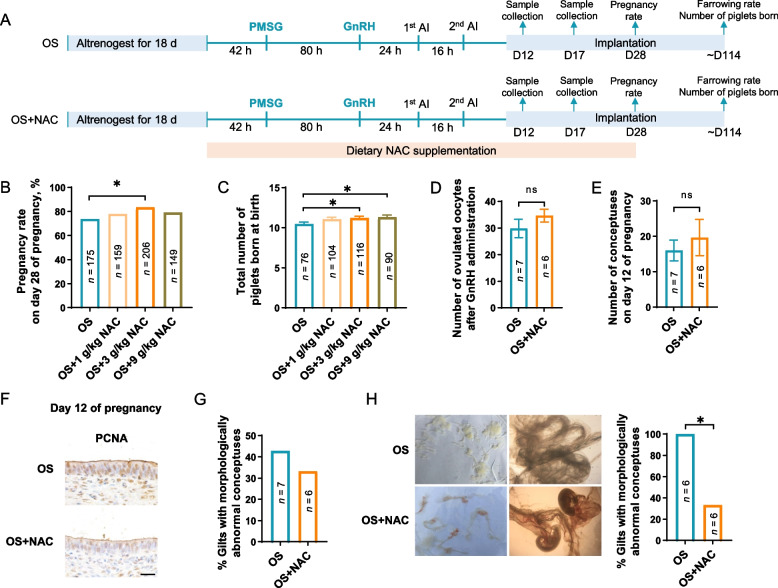
Dietary NAC supplementation alleviates the endometrial overproliferation and enhances pregnancy outcomes in gilts. **A** Schematic illustration of experimental design where gilts were supplemented with or without NAC. **B** and **C** The pregnancy rate (**B**) and litter size (**C**) in gilts supplemented with different doses of NAC. **D** and **E** The number of ovulated oocytes after GnRH administration (**D**) determined by counting the number of corpora lutea, and conceptuses (**E**) on d 12 of pregnancy. **F** Immunohistochemical analysis of PCNA in the endometrium of gilts on d 12 of pregnancy in OS and OS + NAC groups. Scale bar: 20 μm. **G** The percentage of gilts with morphologically abnormal conceptuses on d 12 of pregnancy. **H** The morphology of conceptuses from gilts on d 17 of pregnancy in OS and OS + NAC groups. Right panel: the percentage of gilts with morphologically abnormal conceptuses. Data are presented as the mean ± SEM. *n* value represents the number of gilts. ^*^P < 0.05, ^**^P < 0.01. ns, not significant. OS, ovarian stimulation; NAC, *N*-acetyl-L-cysteine; PMSG, pregnant mare serum gonadotrophin; GnRH, gonadotropin-releasing hormone; AI, artificial insemination; D12, d 12 of pregnancy; D17, d 17 of pregnancy; PCNA, proliferating cell nuclear antigen

Having confirmed the functions of NAC in rescuing endometrial overproliferation, we attempted to test whether NAC can enhance pregnancy outcomes in gilts following ovarian stimulation. To this end, dietary NAC supplementation at various doses was performed until the post-implantation stage (Fig. 6A). We found dietary supplementation of NAC (3 g/kg diet) could increase pregnancy rate and litter size (*P* < 0.05, Fig. 6B and C). Next, we examined the number and morphology of conceptuses on d 12 and 17 of pregnancy when initial adhesion and attachment of elongating conceptuses to uterine luminal epithelium occurs respectively during the peri-implantation stage. Although dietary NAC supplementation did not significantly improve the number of ovulated oocytes and conceptuses on d 12 (*P* > 0.05, Fig. 6D and E), the endometrial epithelial overproliferation induced by ovarian stimulation was greatly reduced (Fig. 6F). In line with the alleviated endometrial overproliferation, NAC supplementation reduced the proportion of gilts that have morphologically abnormal conceptus (Fig. 6G). Similarly, on d 17 of pregnancy, a great proportion of conceptuses displayed visible limb buds and tail buds due to NAC supplementation. In contrast, both developmental delay and complete embryonic loss were evident in the OS group (Fig. 6H).

To further confirm whether NAC improves the pregnancy outcome by enhancing embryonic quality or endometrial receptivity, we used well-established embryo donation model. When blastocysts recovered from NAC-treated donors were transferred to untreated recipient mice, the post-implantation developmental rate was not changed (*P* > 0.05, Fig. S4A). In contrast, when untreated blastocysts were transferred to recipient mice undergoing NAC treatment, the developmental rate was significantly higher than that in untreated recipients (*P* < 0.05, Fig. S4B).

## Discussion

The decrease in pregnancy rate and farrowing rate associated with ovarian stimulation, is of great concern not only for animal reproductive management, but also for human assisted reproduction [2, 8–11]. Our results from the well-established embryo donation model, are in line with the evidence from humans [5], pigs [17] and mice [15], indicate that pregnancy failure associated with ovarian stimulation may be primarily attributed to changes in the uterine environment. In addition, both our own and previously published data [3, 7, 12] indicate that supraphysiological E_2_ levels may be the main factor leading to reduced pregnancy outcomes following ovarian stimulation. Although pregnant pigs show brief increases in E_2_ levels on around d 12 and d 25–30 of pregnancy due to the synthesis by elongating conceptuses [26], which is distinct from that in mice, our analysis of the serum levels of E_2_ suggest that exogenous gonadotropin-controlled ovarian stimulation primarily enhanced ovarian E_2_, but not conceptus-derived E_2_, although the conceptus-derived estrogen has been reported to regulate many endometrial genes during implantation. Thus, we have mainly focused on the enhanced ovarian estrogen, and used porcine endometrial explant model to determine the impact of ovarian derived supraphysiological E_2_ on the uterine receptivity. Actually, it is not surprising that supraphysiological levels of steroid hormones induce morphologic and biochemical endometrial alterations relevant to uterine receptivity. However, despite this, the functional link between the hyperestrogenic state and impaired uterine receptivity following ovarian stimulation, is still not well understood, impeding the creation of a rescue strategy. Previous study in mouse model has indicated that ERɑ mediates proliferative effect of estradiol on the epithelium, by activating downstream FGF-FGFR-ERK signaling cascade [27]. During the window of uterine receptivity, epithelial cells should exit the cell cycle and enter a differentiation phase. Sustained epithelial cell proliferation disrupted uterine receptivity and led to implantation failure [27].

Up to now, an efficient and practical strategy to enhance pregnancy outcomes following ovarian stimulation remains lacking. Minimal-stimulation regimen and FSH step-down regimen [28] have been developed for patients undergoing assisted reproduction to prevent ovarian hyperstimulation. However, both of the regimens resulted in a significantly lower number of oocytes compared to full stimulation. Although reducing excessive estrogen production via aromatase inhibition was thought to be an alternative strategy for improving outcome of treatment after ovarian stimulation [2], previous studies in humans and rodent models [29, 30] indicate a higher incidence of ovarian cyst formation, a delay or complete failure in ovulation due to inappropriate aromatase inhibitor (e.g., letrozole) treatment. These facts also imply that using aromatase inhibitors to ameliorate ovarian hyperstimulation may be considered, but further investigations are needed.

To the best of our knowledge, no effort has been made to improve pregnancy outcomes after ovarian stimulation by directly targeting impaired uterine receptivity. During the establishment of endometrial receptivity, the endometrium undergoes a series of changes, such as the transition from the proliferation to differentiation phase under the control of ovarian steroid hormones [31, 32]. Because the reduction of estrogen-induced endometrial epithelium proliferation is necessary for establishing uterine receptive state before implantation [27], and excessive and prolonged endometrial epithelial cell proliferation is a hallmark defect associated with impaired uterine receptivity [27], we hypothesize if high levels of estradiol disrupted the proliferation of endometrium epithelium. To assess the proliferation status of the endometrium, we used PCNA, a marker expressed during the S phase of the cell cycle. PCNA staining revealed that supraphysiological levels of E_2_ mainly induced proliferation in epithelial cells. Additionally, some PCNA-positive cells exhibited cytoplasmic localization, which may be explained by the nuclear-to-cytoplasmic relocalization during cell differentiation or the possibility of inappropriate intracellular trafficking [33, 34]. The present study identified the pathway that links supraphysiological levels of E_2_ during the preovulatory phase with impaired endometrial receptivity during implantation: hyperphosphorylated FGFR-ERK signaling, is the critical contributor to impaired uterine receptivity. This finding not only provides a mechanistic explanation for reduced fertility after ovarian stimulation, but also offers a potential target for improving pregnancy outcomes following ovarian stimulation.

Targeting the hyperphosphorylated FGFR-ERK signaling, we have tested some candidates that have been reported to inhibit ERK phosphorylation, e.g., melatonin [35, 36], *N*-carbamylglutamate [37] and aspirin [38], neither of which can rescue pregnancy outcomes after ovarian stimulation. Our data, in line with findings from previous studies [39, 40], showed that NAC could efficiently prevent hyperphosphorylation of ERK, thereby reducing overproliferation of endometrial epithelial cells. Of note, NAC has been used to develop a clinical strategy to reduce preterm birth [25, 41], gestational diabetes [42] and polycystic ovarian syndrome [43]. These facts demonstrate that administering NAC would be easy to accept, not only for animal reproductive management, but also for human assisted reproduction.

Our data provided novel insight into how uterine receptivity is regulated in pigs. Despite that our data and the previous study [27] indicating that the critical role of FGF-FGFR-ERK signaling cascade in regulating endometrial receptivity seems conserved among species, their actions may be divergent. Distinct from the paracrine response of epithelial FGFR to FGFs produced by uterine stroma in mice [27], FGF7 has been reported to be specifically abundant in the porcine uterine lumen during the peri-implantation period and regulate epithelial proliferation in an autocrine manner [44]. In addition, our data also update the current knowledge about the role of FGF7 at the maternal–fetal interface in pigs. Previous studies have proposed that FGF7, acting as a paracrine mediator, stimulates the trophectoderm, but not endometrial epithelial cells, to undergo proliferation [20, 45]. In contrast, our study, using an endometrial explant model that underwent exogenous supplementation of FGF7, indicated that FGF7 also stimulates the proliferation of endometrial epithelial cells in an autocrine manner. In addition, we noticed a variable subcellular localization of pERK. Our data and previous studies have shown the cytoplasmic accumulation of pERK in human [46] and porcine uterine [47] epithelial cells, while another study demonstrated the nuclear translocation of pERK in mouse uterine epithelial cells [27], suggesting that the functional pathway of ERK-regulated endometrial epithelial proliferation may be divergent among species. In our results, the FGF7 protein showed both cytoplasmic and nuclear localization in porcine endometrial epithelia, which is in line with a previous report [48]. One reliable explanation for the nuclear localization of FGF ligands is their nuclear trafficking, which may be facilitated by the interplay with other proteins that assist in FGF transportation through the nuclear pores, as well as originating from internalized membrane FGFRs bound to FGF ligands [49]. Another explanation is their interaction with transcription factors or chromatin remodelers that can alter the epigenetic state and transcription of the target genes, especially during the cellular transition between proliferation and differentiation, and possibly responses to hormonal changes during the estrous cycle [50]. These findings imply that FGF signaling may regulate endometrial proliferation not only through the canonical mitogenic effect, but also via chromatin remodeling functions.

## Conclusion

Collectively, our current study, focusing on poor pregnancy outcomes associated with ovarian stimulation, identified that this defect is due to impaired uterine receptivity via hyperactivated FGF-FGFR-ERK signaling cascade; dietary supplementation of NAC can improve the status of endometrial receptivity and enhance pregnancy outcomes. These findings not only fill the long-standing gap between supraphysiological levels of E_2_ during preovulatory phase and impaired uterine receptivity during implantation, but also provide a promising approach to rescue adverse pregnancy outcomes after ovarian stimulation by targeting dysregulated essential signaling in the uterus.

## Supplementary Information

**Additional file 1: Fig. S1** Immunohistochemical image of IgG control. **Fig. S2** Western blotting image of IgG control. **Fig. S3** Impaired uterine environment contributes substantially to the decreased implantation rate after ovarian stimulation. **Fig. S4** NAC increases embryo implantation rate by improving the endometrial receptivity. 

**Additional file 2: Table S1** Summary of primer sequences used for qPCR. **Table S2** Summary of antibodies used for immunohistochemistry and Western blotting.

## Data Availability

All data analyzed during this study are available in the article and/or supporting information, further inquiries can be directed to the corresponding author on reasonable request.

## Abbreviations

CL: Corpora lutea
E_2_: Estradiol
ERK: Extracellular signal-regulated kinase
ESR1: Estrogen receptor 1
FGF: Fibroblast growth factor
FGF7: Fibroblast growth factor 7
FGFR: Fibroblast growth factor receptor
FTAI: Fixed-time artificial insemination
GAPDH: Glyceraldehyde-3-phosphate dehydrogenase
GnRH: Gonadotropin-releasing hormone
hCG: Human chorionic gonadotropin
HOXA10: Homeobox A10
HRP: Horseradish peroxidase
IM: Intramuscular
IP: Intraperitoneal
IVF: In vitro fertilization
mRNA: Messenger RNA
MUC1: Mucin 1
NAC: *N*-Acetyl-L-cysteine
OS: Ovarian stimulation
P_4_: Progesterone
PBS: Phosphate buffered saline
PCNA: Proliferating cell nuclear antigen
pERK: Phosphorylated ERK
pFGFR: Phosphorylated FGFR
PGR: Progesterone receptor
PMSG: Pregnant mare serum gonadotrophin
qPCR: Quantitative real-time polymerase chain reaction
RT: Room temperature
SEM: Standard error of the mean
TBS: Tris buffered saline
