# Supplementary material for: Dietary supplementation with N-acetyl-L-cysteine ameliorates hyperactivated ERK signaling in the endometrium that is linked to poor pregnancy outcomes following ovarian stimulation in pigs
Source: J Anim Sci Biotechnol. 2024 Nov 6;15:148. doi: 10.1186/s40104-024-01109-1 (PMC11539329; doi:10.1186/s40104-024-01109-1)
Supplement: Supplementary file 1 — Additional file 1: Fig. S1 Immunohistochemical image of IgG control. Fig. S2 Western blotting image of IgG control. Fig. S3 Impaired uterine environment contributes substantially to the decreased implantation rate after ovarian stimulation. Fig. S4 NAC increases embryo implantation rate by improving the endometrial receptivity. [file 40104_2024_1109_MOESM1_ESM.docx]

**Dietary supplementation with *N*-acetyl-L-cysteine ameliorates hyperactivated ERK signaling in the endometrium that is linked to poor pregnancy outcomes following ovarian stimulation in pigs**


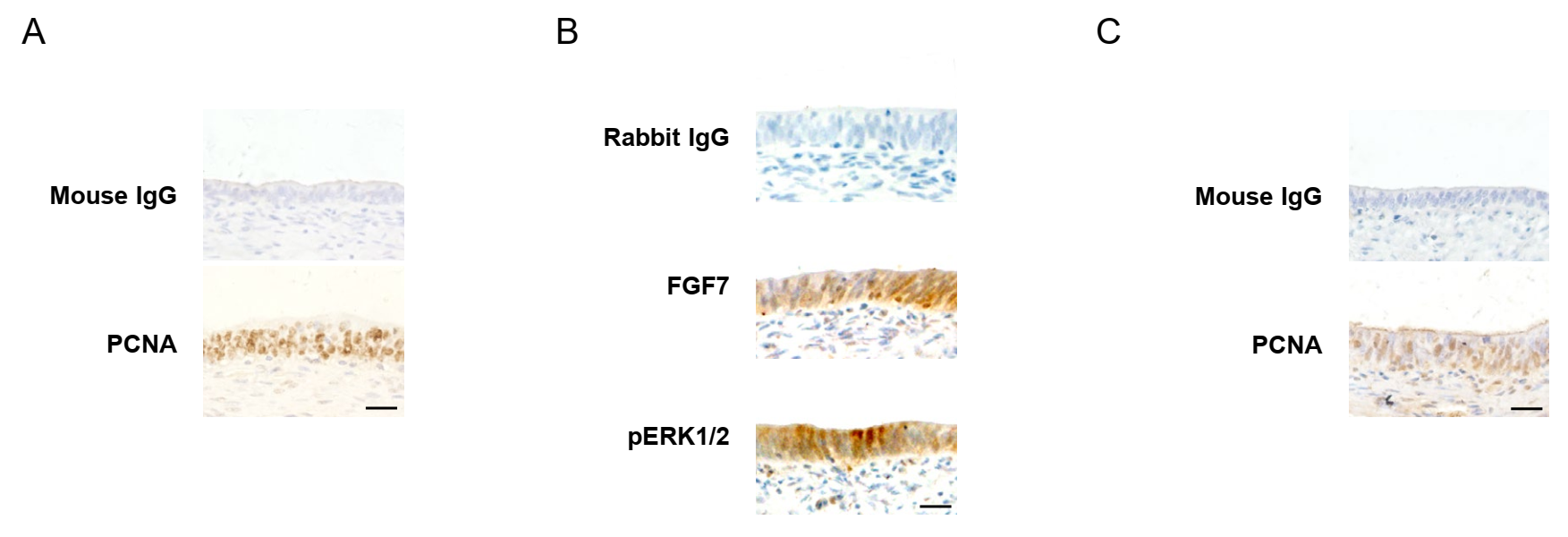


**Fig. S1 Immunohistochemical image of IgG control.**

(A-B) Immunohistochemical image of species matched IgG control in the in vitro cultured porcine endometrial explant. (C) Immunohistochemical image of mouse IgG control in the endometrial tissue from gilt on day 12 of pregnancy. Scale bar: 20 μm.


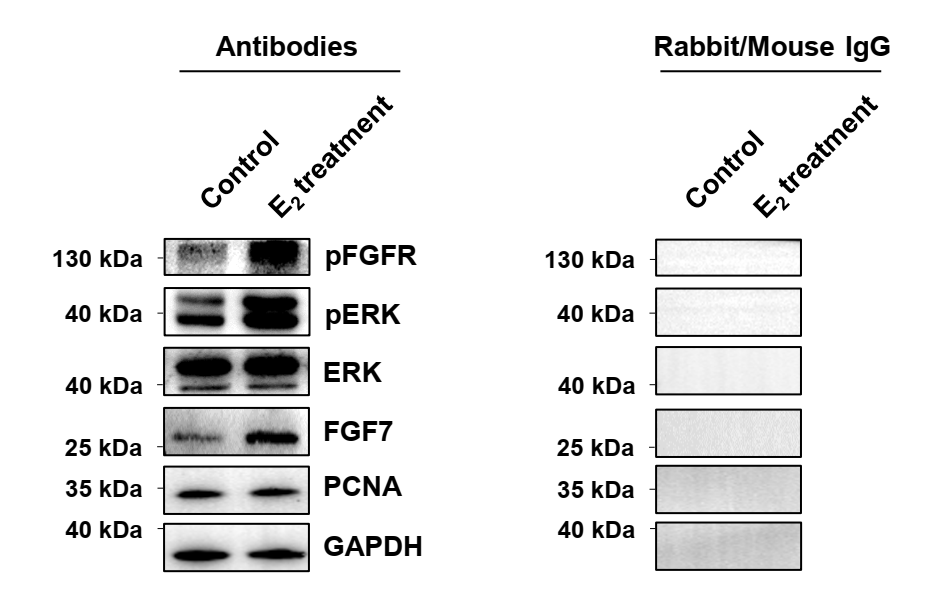


**Fig. S2 Western blotting image of IgG control.**

Western blotting image of IgG control in the in vitro cultured porcine endometrial explants treated with or without E_2_. E_2_, estradiol.


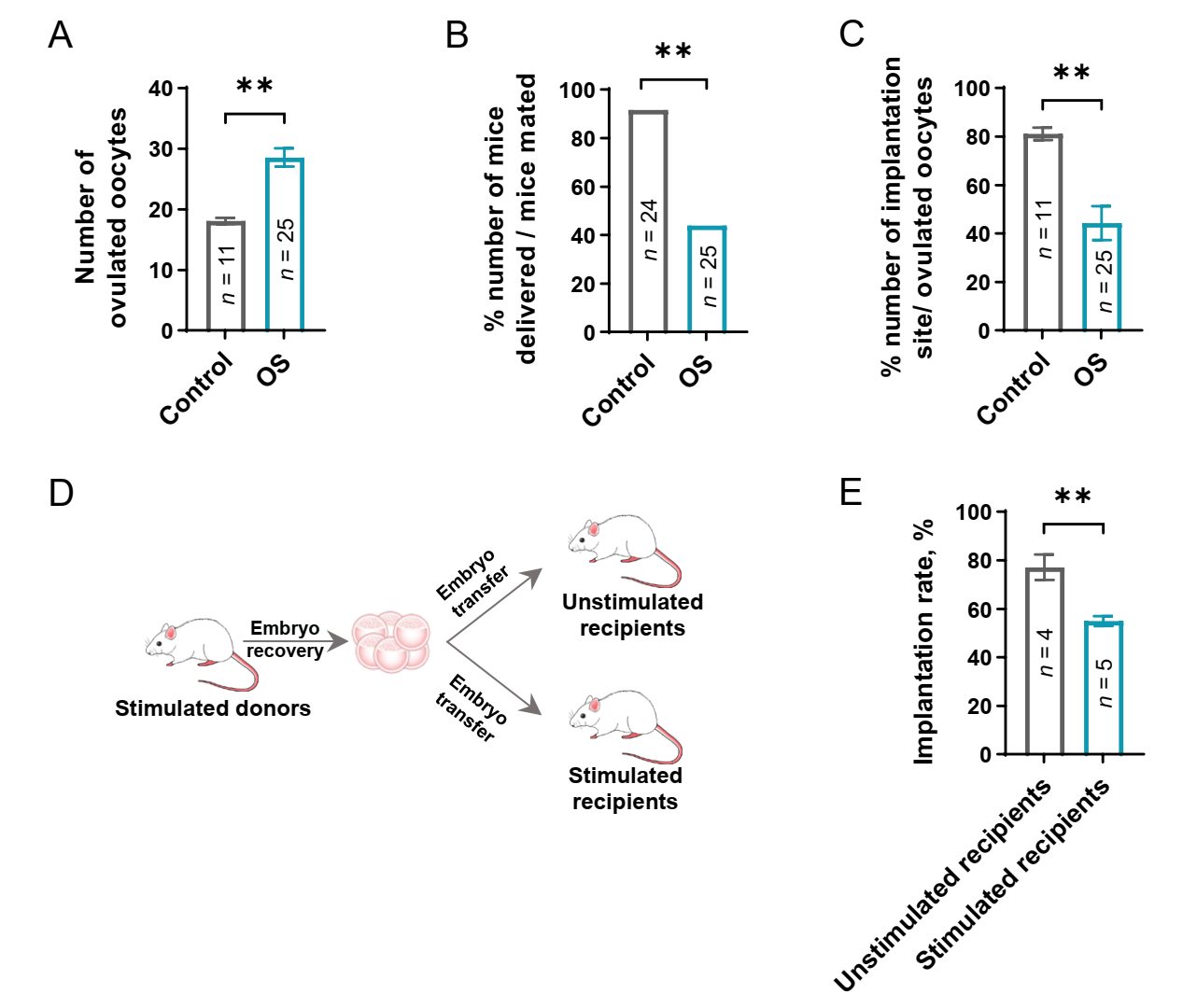


**Fig. S3** **Impaired uterine environment contributes substantially to the decreased implantation rate after ovarian stimulation.**

(A) The number of ovulated oocytes in control and OS groups. (B) The percentage of number of mice delivered to the number of mice mated in control and OS groups. (C) The ratio of the number of implantation sites to the number of ovulated oocytes in control and OS groups. (D) Schematic illustration of experimental design in which embryos recovered from stimulated donors were transferred to recipients treated with or without ovarian stimulation. (E) The implantation rate after embryos were transferred to different recipients. Data are presented as the mean ± SEM. *N* value represents the number of recipient mice. **P* < 0.05, ***P* < 0.01. ns, not significant. OS, ovarian stimulation.


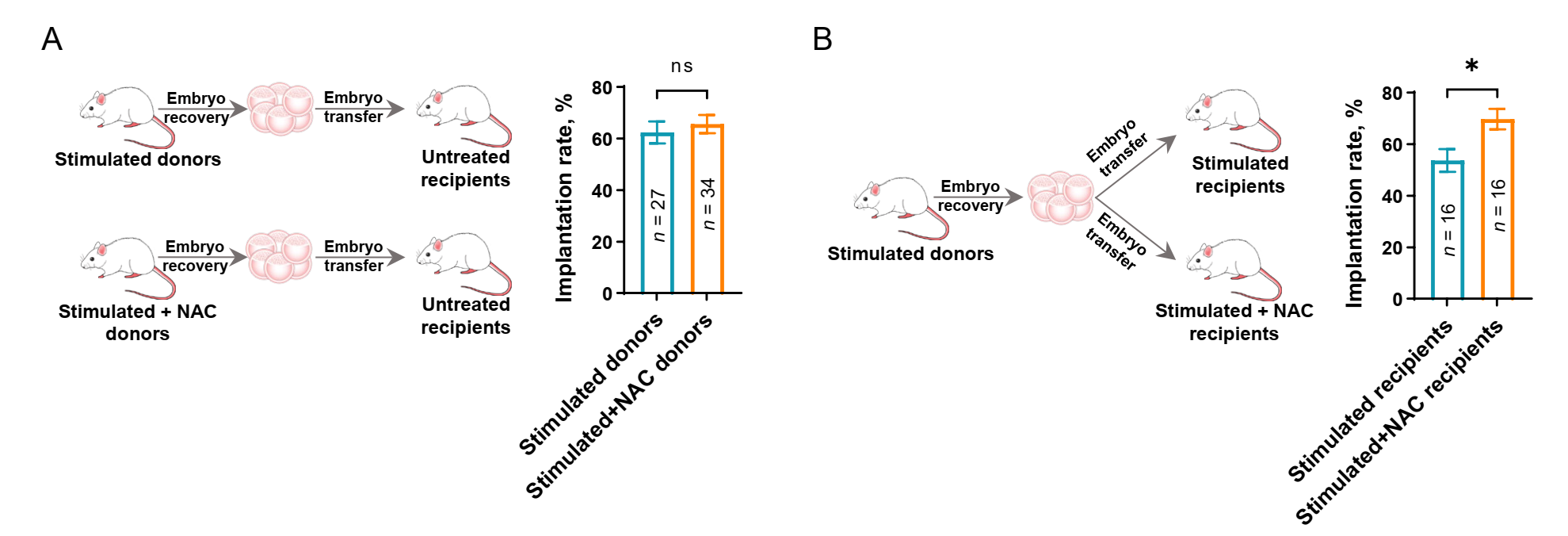


**Fig. S4** **NAC increases embryo implantation rate by improving the endometrial receptivity.**

(A) The implantation rate in unstimulated recipients receiving embryos from stimulated donors treated with or without NAC. Left panel: Schematic illustration of experimental design in which embryos recovered from stimulated donors treated with or without NAC were transferred into unstimulated recipients. (B) The implantation rate in stimulated recipients treated with or without NAC. Left panel: Schematic illustration of experimental design in which embryos recovered from stimulated donors were transferred to stimulated recipients treated with or without NAC. Data are presented as the mean ± SEM. *N* value represents the number of recipient mice. **P* < 0.05. ns, not significant. NAC, *N*-acetyl-L-cysteine.
